# The clinical features and outcome of scan-negative and scan-positive cases in suspected cauda equina syndrome: a retrospective study of 276 patients

**DOI:** 10.1007/s00415-018-9078-2

**Published:** 2018-10-08

**Authors:** Ingrid Hoeritzauer, Savva Pronin, Alan Carson, Patrick Statham, Andreas K. Demetriades, Jon Stone

**Affiliations:** 1 Centre for Clinical Brain Sciences, University of Edinburgh, Edinburgh, UK; 2 Department of Clinical Neurosciences, Western General Hospital, Edinburgh, EH4 2XU UK; 3 Department of Rehabilitation Medicine, NHS Lothian, Edinburgh, UK; 4 Department of Neurosurgery, Western General Hospital, Edinburgh, EH4 2XU UK; 5 Edinburgh Spinal Surgery Outcome Studies Group, Edinburgh, UK

**Keywords:** Cauda equina syndrome, Functional neurological disorder, Psychogenic, Chronic pain, Outcome, Negative scan

## Abstract

**Background:**

The majority of patients presenting with suspected clinical cauda equina syndrome (CES) have no identifiable structural cause for their symptoms (‘scan-negative’ CES). Understanding these patients aids clinical differentiation and management in CES.

**Methods:**

A retrospective electronic note review was undertaken of patients presenting with suspected CES, defined as ≥ 1 of acute bladder, bowel, sexual dysfunction or saddle numbness, to a regional neurosciences centre. We investigated radiology, clinical features, psychiatric and functional disorder comorbidities and outcome of patients with ‘scan-negative’ CES and patients with MRI confirmed compression of the cauda equina (‘scan-positive’ CES).

**Results:**

276 patients were seen over 16 months. There were three main radiologically defined patient groups: (1) ‘scan-positive’ CES (*n* = 78, mean age 48 years, 56% female), (2) ‘scan-negative’ CES without central canal stenosis but with lumbosacral nerve root compression not explaining the clinical presentation (*n* = 87, mean age 43 years, 68% female) and (3) ‘scan-negative’ CES without neural compromise (*n* = 104, mean age 42 years, 70% female). In the two ‘scan-negative’ groups (no neural compromise and nerve root compression), there were higher rates of functional disorders (37% and 29% vs. 9%), functional neurological disorders (12% and 11% vs 0%) and psychiatric comorbidity (53% and 40% vs 20%). On follow-up (mean 13–16 months), only 1 of the 191 patients with ‘scan-negative’ CES was diagnosed with an explanatory neurological disorder (transverse myelitis).

**Conclusions:**

The data support a model in which scan-negative cauda equina syndrome arises as an end pathway of acute pain, sometimes with partly structural findings and vulnerability to functional disorders.

**Electronic supplementary material:**

The online version of this article (10.1007/s00415-018-9078-2) contains supplementary material, which is available to authorized users.

## Introduction

Cauda equina syndrome (CES) is a devastating medical emergency caused by compression of the cauda equina nerve roots which without timely surgery results in bladder, bowel and sexual dysfunction with potential lower limb weakness and numbness [1]. Diagnosis is based on the clinical picture and MRI findings of cauda equina nerve root compression (‘scan-positive’ CES). However, at least half of all patients presenting with the acute clinical CES phenotype (acute bladder, bowel and sexual dysfunction, saddle anaesthesia and pain) have no radiological correlate, so-called ‘scan-negative’ CES. A systematic review of the correlation between history, physical examination and MRI scan result found that the mean prevalence of patients having both clinical and radiological evidence of CES was 14–48% with no single individual sign or symptom being helpful in diagnosing CES [2]; senior neurosurgical trainees asked to predict who would have a positive scan based on history and clinical findings had an accuracy of only 56% [3].

There has been little descriptive study of the ‘scan-negative’ CES group but a better understanding of their presentation may aid clinical differentiation and management. Based on our clinical experience and an initial pilot study of 18 patients from a different centre which demonstrated Hoover’s sign of functional leg weakness in 82% of patients with ‘scan-negative’ CES and 0% of patients with ‘scan-positive’ CES, we hypothesised that some patients with ‘scan-negative’ CES would have evidence of a functional disorder and this may explain at least some of their clinical presentation [4]. By a functional disorder we mean a disorder which is genuine but which is due to an abnormality of nervous system functioning rather than of structure [5]. Functional neurological disorders describe symptoms of abnormal motor and sensory function such as limb weakness or numbness, but does not include chronic pain, even when that is unrelated to a structural cause. Common examples of functional disorders are irritable bowel syndrome and functional neurological disorders. We investigated the radiological findings, demographics, clinical features, comorbidity and outcomes of a retrospective consecutive series of patients referred to a tertiary neurosurgery centre with suspected clinical cauda equina syndrome. Our aims were to better phenotype patients with ‘scan-negative’ CES, to test our hypothesis that at least some patients had evidence of a functional disorder and to generate hypotheses about how functional disorders, medication, pain with or without nerve root compression may interact to explain the bladder symptoms that cause patients to present acutely with ‘scan-negative’ CES.

## Materials and methods

### Definitions

Clinical CES was defined using the Fraser et al. criteria for CES: one or more of bladder, bowel, sexual dysfunction or saddle numbness ± lower limb neurological deficit [1].

Radiological cauda equina compression was defined as > 75% canal stenosis or lack of CSF around the cauda equina nerve roots [6]. ‘Impending’ CES was defined as (a) Fraser et al. clinical criteria, (b) an MRI scan showing a compressive lesion which was large enough to compress the cauda equina nerve roots but which did not meet our radiological criteria and (c) the opinion of the consultant neurosurgeon that the compressive lesion was causing the clinical symptoms and would progress to irreversible CES unless urgently treated.

Patients were defined as with ‘scan-positive’ CES if they had both clinical and radiological evidence of CES or ‘impending’ CES based on the definitions above. Patients were defined as ‘scan-negative’ CES if they satisfied the Fraser et al. criteria, had an urgent MRI scan for possible CES and had no evidence of radiological cauda equina compression on their MRI.

### Methods

#### Recruitment

In July 2016, we carried out a retrospective electronic record review of consecutive referrals with possible cauda equina syndrome to our regional neurosurgery service in Edinburgh between August 2013 and November 2014 with electronic note follow-up until July 2016. Consecutive neurosurgical referrals documented as possible cauda equina syndrome were reviewed manually by two of the authors (IH, SP). Patients were only included in the study if they met clinical criteria for CES. All patients with ‘scan-positive’ CES were included as they were all assessed in the local health board, NHS Lothian, and had clinical symptoms, comorbidities and follow-up outpatient appointments recorded in NHS Lothian. Many patients referred to the neurosurgery service were from other NHS Scotland regions with a different electronic note record which was not possible to access centrally and were not seen in NHS Lothian; these patients were not included. To ensure that clinical data and follow-up were as complete as possible, patients with ‘scan-negative’ CES were only included if referred from an address within the local health board with NHS Lothian documentation of their signs and symptoms or referred via the local health board accident and emergency department. The study received local ethical approval from NHS Lothian (Caldiott Guardian ref 1594).

#### Measures

With respect to the initial admission, all patients had urgent MRI lumbosacral scans which included the cauda equina down to the S5 foramina of the sacrum. A local protocol dictates that a T2 sagittal of cervical and thoracic spine should be done if the MRI lumbosacral spine is normal. All scans were reported by a consultant neuroradiologist.

Using a standardised proforma, we assessed the radiological features, demographics, clinical symptoms and signs, completeness of clinical documentation, and timing of operation (urgent: classified as during the initial admission; elective: classified as after discharge but scheduled due to symptoms and radiology from admission).

We carried out follow-up using electronic records until July 2016 by interrogating scan requests, accident and emergency attendances, all secondary care inpatient and outpatient visits. Information was obtained on functional disorder comorbidity (fibromyalgia; irritable bowel syndrome; chronic fatigue syndrome; non-cardiac chest pain); functional neurological disorders (as defined in DSM 5 including functional motor disorders and non-epileptic seizures); psychiatric comorbidity (such as anxiety/depression/post-traumatic stress disorder (PTSD)/personality disorder/obsessive compulsive disorder(OCD)/suicidal ideation or deliberate overdose/anorexia nervosa); the presence of chronic pain documented in letters; urological symptoms; re-presentations with clinically suspected CES and new diagnoses which explained suspected CES presentation in patients with ‘scan-negative’ CES. When patients had urological symptoms documented during their follow-up, electronic notes were retrospectively reviewed back to 2009 to accurately document the onset of urological symptoms.

#### Statistics

Statistics used were Chi-squared or Fisher’s exact two-sided testing for all symptoms, signs, comorbidities and outcomes. ANOVA was used for comparing mean ages. Statistics were carried out using Statsdirect (http://www.statsdirect.com). All *P* values are comparisons between one of the ‘scan-negative’ groups and the ‘scan-positive’ group.

## Results

276 patients were referred with clinically suspected cauda equina syndrome between August 2013 and November 2014 (Table 1 and Supplementary Figure 1).

### Radiological and demographic findings

During initial admission, seven patients were found to have alternate neurological causes mimicking or causing sacral nerve dysfunction: two patients had evidence of demyelination on MRI of their thoracic cord and both were subsequently diagnosed with multiple sclerosis; two patients had infections causing bladder or sacral symptoms [urinary retention due to urosepsis (*n* = 1) and systemic infection with abscess at L2/3 (*n* = 1)]; three patients had CES mimics [thoracic subdural haematoma (*n* = 1), L1 lumbar fracture (*n* = 1) and metastatic epidural deposit causing thoracic cord compression (*n* = 1)]. We excluded these seven patients from further analysis.

Patients were divided into three main radiological groups:

- 78 had ‘scan-positive’ clinico-radiological CES, including ‘impending’ CES (mean age 48 years (range 21–91), 56% female).
- 87 had ‘scan-negative’ CES but with nerve root compression of at least one nerve root L3–S2 [mean age 43 years (range 20–79), 68% female]. We separated this group on the grounds that some L3–S2 nerve root compression would not have caused sphincter dysfunction but may have impacted on bladder function or promoted functional motor/sensory symptoms in the legs.
- 104 had ‘scan-negative’ CES without neural compromise [mean age 42 years (range 16–81), 70% female].

We will continue with these subdivisions: ‘scan-positive’ CES, ‘scan-negative’ CES with root compression and ‘scan-negative’ CES without neural compression, throughout the rest of the paper.

### ‘Scan-positive’ diagnoses and surgical timing

Of the 78 patients with ‘scan-positive’ CES, 67 (86%) were caused by disc protrusion, the other 11 had various lesions compressing the cauda equina nerve roots: *n* = 4 fractures, *n* = 4 had metastatic deposits, *n* = 1 fracture and a metastasis, *n* = 1 a primary tumour and *n* = 1 large cyst.

68 patients (87%) with ‘scan-positive’ CES had an emergency operation, seven were treated conservatively (*n* = 2 too unwell, *n* = 2 symptoms > 1 week and resolving, *n* = 2 metastatic deposits, *n* = 1 vertebral fracture). In three of these patients sphincter symptoms of CES either turned out to have another cause or resolved but the patients were operated on electively anyway for leg pain.

Sixteen patients with ‘scan-negative’ CES had an operation, 2 urgently, both of whom had nerve root compression and severe pain which did not settle after admission, and 14 electively for leg pain.

**Table 1:** Clinical features of scan-positive and -negative cauda equina syndrome

	Scan +ve (*n* = 78) *n* (%)	Scan −ve with root compression (*n* = 87) *n* (%)	*P* value	Scan −ve no root compression (*n* = 104) *n* (%)	*P* value
Age (mean, SD)	48 years ± 16.8	43 years ± 12.1		42 years ± 12.6	
Gender	56% female	68% female		70% female	
**Operation**					
Emergency	68 (87%)	2 (2%)		0	
Elective	3 (4%)	12 (14%)	< **0.001**	2 (2%)	< **0.001**
**Bladder symptoms**					
Storage problems					
Incontinence	17 (22%)	20 (23%)		42 (40%)	
Urgency/frequency	0	3 (3%)		1 (1%)	
Voiding problems					
Retention	16 (20%)	21 (24%)		26 (25%)	
Reduced awareness	4 (5%)	6 (7%)		3 (3%)	
Hesitancy/difficulty passing	15 (19%)	18 (21%)		11 (11%)	
Mixed problems	0	3 (3%)		11 (11%)	**0.01**
Normal	22 (28%)	15 (17%)		9 (9%)	**0.0005**
**Bowel symptoms**					
Incontinence	6 (8%)	14 (16%)		13 (12%) 1 chronic	
Constipation	11 (14%)	8 (9%)		11 (11%)	
Reduced awareness	1	2 (2%)		2 (2%)	
Normal	27 (35%)	39 (45%)		42 (40%)	
**Sexual function**					
Abnormal	6 (8%)	4 (5%)		2 (2%)	
Normal	0 (0%)	2 (2%)		0	
No info	72 (92%)	81 (93%)		102 (98%)	
**Sciatica**					
Yes	69 (88%)	75 (86%)		80 (77%)	
**Bilateral sciatica**	32 (41%)	17 (20%)	< **0.001**	22 (21%)	**0.001**
No	5 (6%)	7 (8%)		12 (11%)	
Other leg pain	0	1 (1%)		3 (3%)	
**Weakness**					
Yes	35 (45%) (bilateral 13) (17%)	43 (49%) (bilateral = 12) (14%)		52 (50%) (bilateral 19) (18%)	
No weakness	26 (33%)	37 (42%)		36 (35%)	
**Leg numbness**					
Nerve root distribution	48 (61%)	24 (28%)	< **0.001**	38 (36%)	< **0.001**
Bilateral root numbness	18 (23%)	4 (5%)		13 (12%)	
Whole leg	1 (1%)	8 (9%)		9 (9%)	
No numbness	6 (8%)	20 (23%)	**0.01**	17 (16%)	
Non-dermatomal numbness	2 (2%)	16 (18%)	**0.001**	16 (15%)	**0.004**
**Saddle numbness** *	50(64%)	47 (54%)	**0.04**	54 (52%)	**0.02**
Normal	18 (23%)	35 (40%)		42 (40%)	
**Digital rectal exam**					
Reduced anal tone	14 (18%)	18 (21%)		19 (18%)	
Normal	17 (22%)	39 (45%)		44 (42%) 1 refused (1%)	
**Post-void residual**					
< 100 mls	5 (6%)	14 (16%)		12 (9%)	
> 100–500 mls	7 (9%)	11 (13%)		5 (5%)	
> 500 mls	3 (4%)	2 (2%)		6 (6%)	
No info	63 (81%)	60 (69%)		81 (78%)	

Bold—*P* values of < 0.05 were deemed significant

*P* values refer to comparison against scan-positive group and are only shown if significant

*SD* standard deviation

*Saddle numbness: as assessed by pin prick sensation

### Clinical features

Urinary function (*n* = 263, 98%), lower limb pain (*n* = 250, 93%) saddle sensation (*n* = 247, 92%), lower limb power (*n* = 229, 85%) and sensation (*n* = 225, 84%) were often documented. Bowel function (*n* = 177, 66%), anal tone from digital rectal examination (*n* = 151, 56%) and sexual function (*n* = 14, 5%) were poorly or very poorly documented.

#### Symptoms

Patients with scan-positive CES were more likely to have symptoms of bilateral sciatica and, surprisingly, were less likely to have documented bladder dysfunction than patients in either of the ‘scan-negative’ CES groups (see Table 1). These are two controversial findings so we reviewed them in detail. Even when both ‘scan-negative’ groups were combined bilateral sciatica was still significantly more likely in patients with ‘scan-positive’ CES’ (38% vs. 20%, *n* = 30/78 vs. *n* = 39/191, *P* = 0.002). The patients with normal bladder function met our criteria as ‘impending’ cauda equina syndrome. These patients all had radiological evidence of cauda equina compression and one or more other signs of clinical cauda equina syndrome, most commonly saddle numbness, documented in twenty patients or bowel or sexual dysfunction in four patients each.

#### Signs

Patients with ‘scan-positive’ CES were more likely to have saddle numbness (64% vs 54% and 52%, *P* = 0.04, 0.02), although rates were relatively high (> 50%) in all groups.

### Comorbidity functional and psychiatric disorders

Both patient groups with ‘scan-negative’ CES were more likely to have a comorbid functional disorder, functional neurological disorder and psychiatric diagnoses than patients with ‘scan-positive’ CES when assessed at follow-up in July 2016 (see Table 2). The specificity of finding a comorbid functional neurological disorder in ‘scan-negative’ CES at presentation was 1 (0.95–1) although sensitivity was low, 0.09 (6–14).

**Table 2:** Functional and psychiatric comorbidity in scan-positive and -negative cauda equina syndrome

	Scan +ve (*n* = 78) *n* (%)	Scan −ve with root compression (*n* = 87) *n* (%)	*P* value	Scan −ve no root compression (*n* = 104) *n* (%)	*P* value
**Functional disorder comorbidity**	7 (9%)	26 (30%)	**0.0007**	39 (37%)	< **0.0001**
Functional disorders*					
Irritable bowel syndrome	2 (3%)	9 (10%)		12 (11%)	
Non-cardiac chest pain	0	7 (8%)		17 (16%)	
Chronic widespread pain	5 (6%)	5 (6%)		8 (8%)	
Other		1 atypical facial pain		2 functional cognitive disorder	
Functional neurological disorders*	0	10 (11%)	**0.0014**	13 (12%)	**0.0005**
Limb weakness		3 (3%)		6 (6%)	
Sensory/hemisensory		4 (5%)		5 (5%)	
Dissociative seizures		2 (3%)		1 (1%)	
Other		2 (2%) Dysphonia		2 (2%) Visual	
Psychiatric diagnoses*					
Depression	17 (22%)	34 (39%)	**0.02**	55 (53%)	< **0.0001**
Anxiety	14 (18%)	26 (30%)		43 (41%)	
Personality disorder	8 (10%)	21 (24%)		17 (16%)	
Other	0	2 (2%) 1 anorexia 1 OCD 1 suicidal ideation		1 (1%) 3 (3%) PTSD 2 deliberate overdose	
**Timing of FND in relation to CES presentation**					
Prior		6 (7%)		6 (6%)	
At the same time		2 (2%)		4 (4%)	
After		2 (2%)		3 (3%)	

Bold—*P* values of < 0.05 were deemed significant

*FND* functional neurological disorder, *OCD* obsessive compulsive disorder, *PTSD* post-traumatic stress disorder

*Several patients had more than one disorder

### Outcomes: pain, re-presentation rate, and bladder function

There were no significant differences between the three groups in the follow-up frequency (93% vs. 89% and 87%) or mean duration of follow-up (average 13 months, 16 months, 16 months) (Table 3).

Only one patient in the scan-negative groups presented at follow-up with an alternative neurological explanation for CES. This patient had transverse myelitis. They had no comorbid functional disorders.

Four patients with ‘scan-positive’ CES (4%) re-presented during the study time with a new episode of clinical cauda equina syndrome, two of whom required re-operation. Re-presentations with possible cauda equina syndrome necessitating an urgent scan during follow-up occurred in 22 of the 191 patients (11%) with ‘scan-negative’ CES, all of whom continued to have negative scans. Fifteen patients re-attended once, five re-attended twice and two patients re-attended three times (see breakdown in Table 3). Only 5 patients (23%) re-presented within 1 month suggesting their recurrent presentations related to 1 episode of ongoing symptoms, the other 17 presented over a longer period suggesting multiple different episodes of symptom occurrence.

Patients with ‘scan-negative’ CES in both groups were more likely to have chronic pain recorded in the electronic patient record on follow-up (26% vs 58% and 59%).

Rates of bladder dysfunction in the electronic patient record were not significantly different in all groups. Pre-existing bladder symptoms were found in two patients with ‘scan-positive’ CES, one patient within the ‘scan-negative’ CES with root compression group and in four patients in the ‘scan-negative’ without neural compression group. One patient from each group had prior episodes of urinary retention. After CES presentation, idiopathic urinary retention affected one person in the ‘scan-negative’ with root compression group and three patients without neural compression.

**Table 3:** Follow-up and outcomes

	Scan +ve (*n* = 78) *n* (%)	Scan −ve with root compression (*n* = 87) *n* (%)	*P* value	Scan −ve no root compression (*n* = 104) *n* (%)	*P* value
Average follow-up/months	13	16		16	
No follow-up	10 (13%)	6 (7%)		12 (11%)	
Deceased or palliative	4			1	
Cause of clinical CES found	100%	0		1 (1%)	
**Re-presentation with clinical CES**					
Once	3 (4%)	10 (11%)		12 (11%)	
Twice	3 (4%)	8 (9%)		7 (7%)	
Three times	N/A	2 (2%) 2 (2%)		3 (3%) 2 (2%) 3 (3%)	
Prior ‘scan-positive’ CES	2 (3%)	3 (3%)		2 (2%)	
Prior ‘scan-negative’ CES		6 (7%)		9 (9%)	
Chronic pain	20 (27%)	52 (60%)	< **0.0001**	60 (58%)	< **0.0001**
**Bladder disorders**					
Total affected	8 (10%)	8 (9%)		11 (11%)	
Storage problems					
Neurogenic bladder	7 (9%)	0		0	
Overactive bladder	1 (1%)	1 (1%)		1 (1%)	
Stress incontinence		1 (due to prolapse)		0	
Urge incontinence		0		2 (2%)	
Voiding problems					
Idiopathic urinary retention		1 (1%)		3 (3%)	
Urethral stenosis		1(1%)		2 (2%)	
BPH		1 (1%)		1(1%)	
Other		1 (1%)UTI 2 idiopathic haematuria		1 bladder outlet obstruction 1 enuresis	
**Timing of urological diagnoses** **Before CES presentation**					
Stress urinary incontinence	2 (3%)	1 (1%)		4 (4%)	
Urge incontinence	1 (1%)			2 (2%)	
Idiopathic urinary retention		1 (1%)		1 (1%)	
Bladder outlet obstruction	1 (1%)			1 (1%)	
At time of diagnosis	0	1 (1%) UTI		0	
After CES presentation	6 (8%)	6 (7%)		7 (7%)	

Bold—*P* values of < 0.05 were deemed significant

## Discussion

We found that patients with ‘scan-positive’ and ‘scan-negative’ CES presented with similar core symptoms. Saddle anaesthesia and bilateral sciatica with radicular sensory abnormalities were common in patients with ‘scan-positive’ CES, whilst non-dermatomal sensory loss and mixed urinary problems were more commonly seen in patients with ‘scan-negative’ CES. However, as in previous studies, no individual clinical symptom or sign could accurately differentiate between scan-positive and ‘scan-negative’ CES [7]. The explanation for scan-negative CES does not appear to be latent neurological disease, of which there are many causes (Table 4) [8–19], at least in the majority of patients, since we only found one patient where this was the case at follow-up.

The neurological differential diagnoses for ‘scan-negative’ CES were considered by the authors and encompasses inflammatory, infectious, vascular, neoplastic and neurodegenerative disorders (Table 4). In some cases, these conditions can be difficult to diagnose and may present initially as peripheral disorders but are caused by central mechanisms. This is particularly the case in patients with arteriovenous malformations including dural AV fistula [15]. Patients may present several times prior to diagnosis but symptoms are progressive and ultimately upper motor neurone signs appear. Transient infectious causes of lumbosacral polyradiculitis, such as Elsberg syndrome, caused by HSV, may also be difficult to pick up as lumbar puncture results normalise quickly and can have poor positive predictive value [12]. In a recent study at the Mayo clinic, five patients over a 16-year period were felt to have Elsberg syndrome causing cauda equina radiculitis [12]. Bladder symptoms affect approximately 75% of patients with multiple sclerosis and are often cited as one of the most unpleasant symptoms by patients [8]. However, it is unusual for patients to present with bladder symptoms only and the diagnosis of multiple sclerosis is based upon clinical events and lesions separated in time and space.

In keeping with our hypothesis, patients with ‘scan-negative’ CES did have notably more functional somatic disorders, psychiatric comorbidity and especially functional neurological disorders than patients with scan-positive CES who had similar sphincter and leg symptoms. The specificity for functional neurological disorders in this scenario for ‘scan-negative’ CES was 1 (0.95–1) although sensitivity was 0.12 (7–17) with around half of patients developing their functional neurological disorders during their ‘scan-negative’ CES presentations (Table 2).

The data support our earlier pilot study and strongly suggest that at least some patients with ‘scan-negative’ CES have symptoms due to acute functional limb weakness, numbness and functional, pain or medication-related urinary symptoms. Our findings are in keeping with other studies showing functional neurological disorders are commonly triggered by pain. For example, a systematic review of 869 patients with functional motor and sensory symptoms found that physical injury preceded onset in 37% cases [20, 21]. In the last 10 years, the understanding and awareness of functional neurological disorders has increased significantly. Diagnosis is made on the basis of positive clinical signs, such as Hoover’s sign of functional leg weakness—weakness of hip extension which normalises with contralateral hip flexion, which has good diagnostic sensitivity and specificity [22]. A positive diagnosis and tailored physiotherapy seems to be more effective for functional motor disorder than standard treatment with 72% of patients improving in a recent randomised trial compared to only 18% of the control group [23]. Understanding of the mechanism of functional neurological disorders has expanded from Freudian ideas of conversion to Bayesian ideas of ‘top-down’ expectation and abnormal self-directed attention overriding the normal sensory and motor pathways [24, 25].

Psychiatric disorders are not uncommon in the population; however, levels of 40 or 50% are higher than would be expected even in patients with chronic neurological disease [26] and in higher than psychiatric comorbidity in some studies of patients with chronic back pain [27]. Patients with avoidance and panic are more likely to develop chronic pain so knowledge and appropriate treatment of these comorbidities are important [28]. Urological symptoms requiring urology input were similar in both groups. This is noteworthy given that urological symptoms are one of the most common reasons why patients with ‘scan-positive’ CES must be urgently operated on. High numbers of patients in the ‘scan-negative’ groups represented with clinical CES requiring an urgent scan which was always negative. This suggests that not only are patients having recurrent symptoms which correlate with clinical CES, as per the Fraser et al. criteria, but that they are also high-resource users and we should make more effort to understand and treat them.

### Hypothetical mechanisms for ‘scan-negative’ CES

The excess of abnormal bladder symptoms in the patients with ‘scan-negative’ CES was of particular interest and potentially counters many clinicians’ expectations. There are several possible hypotheses about the origin of bladder symptoms in patients with ‘scan-negative’ CES. First, pain causing sympathetic hyperactivity and increased inhibitory signals via the hypogastric and pelvic nerves could be resulting in increased contraction of the internal urethral sphincter and override normal voiding parasympathetic processes causing difficulty voiding. Second, pain or panic may have exacerbated underlying bladder dysfunction including incontinence which occurs in up to one-fifth of middle-aged women [29] and is more common in patients with anxiety and depression [30] or chronic back pain [31]. Third, analgesic medications have significant effects on the bladder. Medications such as pregabalin, gabapentin and benzodiazepines can cause or exacerbate urinary incontinence [32, 33]. Opiates are well known to affect the bowels but the effect on the bladder, which if severe can lead to chronic urinary retention, is less well recognised [34]. Opiates can also cause severe constipation and there is a case report of constipation causing pelvic nerve entrapment and mimicking cauda equina syndrome [35]. From the authors’ experience, it is much more common that patients are constipated from medications and this results in more pain and difficulty passing a bowel motion. Fourth, a cause of chronic urinary retention triggered by pain or medications is Fowler’s syndrome, which describes primary failure of the external urethral sphincter to relax. Patients with Fowler’s syndrome have high rates of chronic pain and functional neurological disorder comorbidity [36]. Fowler’s syndrome has detectable neurophysiological changes and its aetiology remains uncertain but one possibility is that it represents a primary functional disorder of the urethral sphincter and a chronic model of the type of retention or voiding dysfunction seen in some patients with scan-negative cauda equina. Finally, previous studies of patients presenting for routine lumbar decompression found bladder symptoms in 55% [37] and an additional urodynamics study of a similar patient group found 26% had urodynamic evidence of detrusor areflexia all of whom reported abdominal straining to void [38]. This may be due to downstream effects of compression or inflammation from higher nerve roots; however, there was only one patient with idiopathic urinary retention in the ‘scan-negative’ with root compression group on follow-up, so this explanation seems unlikely to be a major cause of symptoms in the ‘scan-negative’ groups.

Considering these ideas, we propose that at least some patients with scan-negative CES patients can be best understood to have a functional disorder explaining some, or all, of their presentation. We hypothesise that many patients have a vulnerability either to functional disorder and/or a prior underlying bladder dysmotility disorder. In some cases, patients may respond to severe back muscle spasm or pain from disc herniation and nerve root entrapment with panic and dissociation > resulting in either inability to contract the pelvic floor causing incontinence or inability to relax the pelvic floor and urethral sphincter causing urinary retention. Acute or long-term analgesia such as opiates may cause further retention, or gabapentinoids may cause incontinence, worsening the bladder dysfunction. Patients then present to hospital with clinical CES where they typically receive reassurance (although no explanation for why they had sphincter symptoms), pain relief and physiotherapy. However, for the 50% who develop chronic pain and the 11% who have recurrent episodes of suspected CES, fear of movement and an attentional focus of symptoms may lead to deconditioning and a centrally generated pain syndrome with consequent inability to return to normal activity.

### Limitations

The retrospective nature of the study and its dependence on electronic notes resulted in missing data. The design means that data about clinical features were not collected through routine practice and not systematically. This may explain our potentially controversial findings of bilateral sciatica being more common in patients with ‘scan-positive’ CES, although we think this is unlikely, especially given the high rate of symptom documentation (95%). Patients with ‘scan-positive’ CES, including those with ‘impending’ CES, were more likely to have normal bladder function than patients with ‘scan-negative’ CES which also was an unexpected finding of our study. The high frequency of missing data about sexual function was surprising and may be important in differentiating ‘scan-positive’ from ‘scan-negative’ CES. Not all patients with normal radiology saw a neurologist, for example, if they were discharged quickly. This means that functional comorbidity may have been underestimated. All patients with ‘scan-positive’ CES from South East Scotland were included whereas only ‘scan-negative’ CES patients from a smaller area (NHS Lothian) with complete medical records were included; hence, this study cannot be used to estimate CES incidence or compare incidence of ‘scan-positive’ vs. ‘scan-negative’ CES. However, this limitation means that scan-negative CES is likely to be *even more* common than we have demonstrated in this study. Medication records were not accurate enough for inclusion in the study and this is a gap in the data. Primary care data about outcome on follow-up were not available and this may lead to an underestimation of urological or pain symptoms during follow-up in all groups. Some additional neurological diagnoses may have been missed; however, our departmental policy of a T2 sagittal MRI of the thoracic and cervical spine for lumbosacral scan-negative CES identified seven patients who immediately obtained a non-CES diagnosis. Only one additional diagnosis was found on follow-up at 16 months with 88% follow-up, and among the 22 patients who re-attended and were investigated again for ‘scan-negative’ CES, no new diagnoses were made. This suggests that alternative neurological diagnoses are unlikely to explain a high proportion of scan-negative CES. We believe immediate investigation and diagnosis is one of the reasons there was only one new diagnosis at follow-up.

## Conclusion

We found that of 276 consecutive CES patients, 28% (*n* = 78/276) were ‘scan positive’, 69% (*n* = 191/276) were ‘scan negative’ and 3% (*n* = 7/276) had an alternative cause mimicking or causing sacral nerve dysfunction. There was no single clinical feature which differentiated between the groups. Of the scan-negative patients, just under half of patients had a nerve root compression that may have contributed but did not explain their clinical presentation. These patients with ‘scan-negative’ CES were more likely to have comorbid psychiatric and functional disorders and have chronic pain on follow-up. The data support a model in which ‘scan-negative’ cauda equina arises as an end pathway of acute pain, sometimes with partly structural causes, medication side effects and vulnerability to functional disorder. A prospective study with systematically collected clinical data, additional imaging and neurological assessment would reduce these limitations.

**Table 4:** Uro-neurological differential diagnoses of clinical cauda equina syndrome with normal MR imaging

	Urinary retention	Urinary incontinence
Neurological differential diagnoses*		
Inflammation	Myelitis	Multiple sclerosis [8] Myelitis especially neuromyelitis optica spectrum disorder [9]
Infectious	Elsberg’s syndrome [12], varicella zoster, cytomegalovirus, herpes simplex, HIV [13, 14]	
Vascular	Arteriovenous malformation [15], spinal infarction [16]	Cerebral stroke [17]
Neoplastic	Neoplastic or radiation induced [18]	
Neurodegenerative	Multiple system atrophy [19]	Parkinson’s disease [19]
Urological differential diagnoses	Fowler’s syndrome [10] Idiopathic urinary retention	Exacerbation of prior urinary incontinence (affects 20% women over 40) [29] Bladder pain syndrome [11]
Medications (side effects recorded from the British National Formulary)	Opiates Anticholinergics (e.g. tricyclics) Benzodiazepines NSAIDs (risk increases in elderly and with higher doses)	Benzodiazepines Pregabalin SSRIs ACE inhibitors/diuretics
Other possibilities	Pain: radiculopathy is a common comorbidity Many cervico/thoracic pathologies can lead to cauda equina symptoms	

## Electronic supplementary material

Below is the link to the electronic supplementary material.

Supplementary material 1 (DOCX 58 KB)
