# Supplementary material for: The clinical features and outcome of scan-negative and scan-positive cases in suspected cauda equina syndrome: a retrospective study of 276 patients
Source: J Neurol. 2018 Oct 8;265(12):2916–26. doi: 10.1007/s00415-018-9078-2 (PMC6244667; doi:10.1007/s00415-018-9078-2)
Supplement: Supplementary file 1 — Supplementary material 1 (DOCX 58 KB) [file 415_2018_9078_MOESM1_ESM.docx]

| **Supplementary Table One:** Total ‘scan positive’ CES group vs. combined ‘Scan negative’ CES groups | | | |
| --- | --- | --- | --- |
|  | Scan positive CES (n=78) | Scan negative CES (n=191) | Significance;  two sided Fisher’s exact test  (p<0.05) |
| Weakness | (37/60 recorded) 61% | (99/171 recorded) 58% | 0.7 |
| Numbness | (49/ 52 recorded) 94% | (111/148 recorded) 75% | 0.002 |
| Urinary symptoms | (57/74 recorded) 77% | (165/189 recorded) 87% | 0.05 |
| Bowel symptoms | (25/45 recorded) 77% | (51/131) 39% | 0.06 |
| Bilateral sciatica | (31/73) 40% | (39/190) 20% | 0.005 |
| Saddle numbness | (50/66) 76% | (102/179) 60% | 0.007 |
| Functional comorbidity | 5 (6%) | 62 (32%) | 0.0001 |
| - Of which Functional Neurological Disorder | 1% (1%) | 21(11%) |  |
| Psychiatric co morbidities | (17/78) 22% | (88/191) 46% | 0.0002 |
| Outcome: chronic pain | (20/78) 26% | (97/191) 51% | 0.0001 |

Supplementary Table Two: Investigation of Urological Symtpoms

| **Investigations** | **Scan +ve**  **(n=78)**  **Total n=8** | **Scan -ve with root compression (n=87)**  **Total n=8** | **Scan -ve no root compression (n=104)**  **Total n=11** |
| --- | --- | --- | --- |
| **Post void residual** |  |  | **1** |
| **Gynaecology Review*** |  | **1** | **4** |
| **Urology Review*** |  | **1** | **2** |
| **Trail removal of catheter** | **1** |  |  |
| **Urethrogram** | **1** |  |  |
| **Uroflowmetry** | **2** | **2** |  |
| **Cystoscopy** |  | **3** | **2** |
| **Urodynamics** | **1** |  | **2** |
| **Video-urodynamics** | **2** |  |  |
| **Other** | **1 no information but referred to urology** | **1 UTI diagnosed by neurosurgical team** |  |
| ***= no additional investigations** | | | |

Supplementary Figure One
